# A Joint Measurement and Sensitivity Enhancement Method for Improving EIT Image Reconstruction Quality

**DOI:** 10.3390/s26216830

**Published:** 2026-10-29

**Authors:** Yanlong Li, Xiaonan Wei, Bowen Xu, Honghao Qu, Shihong Yue

**Affiliations:** 1 CCCC Tianjin Dredging Co., Ltd., Tianjin 300461, China; liyanlong@ccccltd.cn (Y.L.); xiaonanwei@ccccltd.cn (X.W.); xubowen@ccccltd.cn (B.X.); 2 School of Electrical and Information Engineering, Tianjin University, Tianjin 300072, China; quhh@cnmstc.com

**Keywords:** electrical impedance tomography, target reconstruction, measurement enhancement, sensitivity enhancement, iterative optimization

## Abstract

Electrical impedance tomography (EIT) is widely used in industrial and biomedical applications, but weak central sensitivity can obscure internal targets. This study proposes a joint enhancement (JE) method combining center-residual feedback with regional sensitivity-based measurement enhancement. Reconstructed peripheral structures are used to approximate unavailable target-free measurements and iteratively extract central-target residual information. Regional sensitivity decomposition enhances the central measurement component, which is reconstructed using Tikhonov regularization and integrated with the peripheral structure. Tests on five simulated thoracic models and five saline-tank configurations show that JE achieves the highest correlation coefficient (CC) and lowest relative error (RE) among the four compared methods. Relative to the initial linear back-projection reconstruction, JE increases mean CC by approximately 15.48% and reduces mean RE by 25.87% in simulation; the corresponding experimental improvements are 21.68% and 12.82%, respectively. These results support improved central-target recovery for the tested configurations. The proposed method as a whole has the potential to be applied to general EIT processes.

## 1. Introduction

Tomographic imaging plays an important role in practice [1,2,3]. Unlike the most widely used computed tomography (CT) [4], electrical impedance tomography (EIT) [5] is a visualization technique that does not use ionizing radiation. According to the boundary measurements at a detection field, a target reconstruction algorithm can estimate the internal target distribution in the field [6].

Despite substantial progress, at least the following issues remain unresolved:

1. *Sensitivity difference*. EIT boundary sensitivity is generally higher than that in the center of the field, resulting in poor recovery of central targets [7]. Direct use of the raw measurements does not adequately reveal the intrinsic relationship between target changes and their boundary measurements.
2. *Uncertain measurements*. Due to random and complex measuring conditions, electrode contact, object variations, fluctuations in the background medium, and noise all affect the EIT measurements. The reliability of individual measurement components therefore varies, reducing reconstruction accuracy [8].
3. *Local sensitivity*. Targets usually appear as one or a few localized changes. Because the sensitivity matrix is central to EIT reconstruction, increasing sensitivity at a target location is desirable for improving detectability [9,10]. Nevertheless, most existing methods cannot readily find all targets, even when a positional prior is available. Regularization is also required to stabilize EIT reconstruction. Classical Tikhonov regularization (TR) [11] suppresses instability through a penalty term, but a single global parameter cannot accommodate spatially heterogeneous changes and does not provide a general mechanism for adaptively partitioning the imaging field.

To address these issues, EIT research has gradually moved from isolated improvements in reconstruction algorithms toward the integration of measurement-feature enhancement, measurement-reliability assessment, adaptive sensitivity correction, and model-data integration [12]. Laganà et al. [13] compared adjacent and opposite excitation schemes in three-dimensional finite-element tissue models and found that opposite excitation improved impedance contrast, spatial sensitivity, and the localization of neoplastic inclusions. Recent comparative studies [14] further show that deep-learning and model-driven approaches can markedly improve EIT reconstruction, but their performance depends strongly on the training distribution. When lesion position, size, or conductivity contrast falls outside that distribution, reconstruction quality may deteriorate, making hybrid physics- and data-driven methods an important direction. These approaches, however, do not fundamentally remove the loss of small central targets caused by high peripheral sensitivity and low central sensitivity. The dual-branch structure-aware feature fusion network (DSFF-Net) [15] fuses anatomical priors with conductivity features from structural segmentation and conductivity reconstruction branches to improve three-dimensional EIT imaging for pediatric pneumonia. Its improved accuracy and robustness in simulated and clinical tests highlight the value of structural priors, while its use of CT-derived training data distinguishes it from the measurement-based enhancement proposed here. The Deep Dynamic Image Prior (D2IP) framework [16] combines unsupervised parameter warm-start, temporal parameter propagation, and a lightweight reconstruction backbone for training-data-free three-dimensional time-sequence pulmonary EIT. Its gains in image quality and computational efficiency demonstrate the value of temporal and architectural priors, complementing the present work’s focus on enhancing sensitivity to small central targets.

For uncertain measurement conditions, research has progressed from simple filtering toward methods for identifying and correcting electrode displacement, evaluating signal quality in real time, and correcting subject-specific thoracic models. Compensation for electrode position and boundary variation can reduce reconstruction errors caused by model mismatch [17]. Clinical lung-EIT studies also emphasize electrode–skin contact impedance, respiratory motion, electrode displacement, and signal-quality control; consensus recommendations identify stable contact and reliable boundary voltages as prerequisites for trustworthy EIT images [18]. In practice, however, contact variation, field deformation, background-conductivity changes, and random noise often occur simultaneously and interact. Their effects differ among voltage components, and a unified method that adaptively evaluates component reliability and adjusts reconstruction contributions accordingly remains unavailable.

Sensitivity-matrix and regularization research has developed from fixed matrices and a single TR parameter toward sensitivity weighting, spatially adaptive regularization, total-variation constraints, and sensitivity correction. Spatial priors or initial reconstructions can impose different constraints in different regions, improving resolution and reducing oversmoothing [19]. Regional partitioning, clustering, and other prior information have also been used to construct local sensitivity models and to update sensitivity or regularization weights using measurement residuals [20]. Most existing methods, however, still rely on global correction, offline calibration, or posterior partitioning derived from an initial image; they mainly improve overall image quality rather than actively strengthening sensitivity in a suspected lesion region. Posterior partitioning can also fail when the lesion is small, lies in a low-sensitivity central region, or is absent from the initial reconstruction. It therefore remains important to extract informative local-abnormality features from measurements, account for component reliability, identify candidate central regions, and selectively enhance the corresponding local sensitivity and regional regularization.

This study investigates two complementary strategies for improving EIT target identification, particularly for weak central targets. First, a center-residual feedback (CRF) procedure approximates the unavailable target-free measurements from reconstructed peripheral structures and iteratively updates the residual to reveal central-target information. Second, regional sensitivity contributions are used to decompose the residual and enhance its central component. Linear back-projection (LBP) [21] provides rapid intermediate reconstructions without an additional regularization parameter, while TR stabilizes the final reconstruction. The enhanced central component is then combined with the peripheral structure to obtain a complete image. Together, these strategies aim to improve central-target visibility and localization while preserving the overall spatial structure of the conductivity distribution.

## 2. EIT Principles and Reconstruction Fundamentals

EIT includes electrical resistance tomography (ERT) [22], electrical capacitance tomography (ECT) [23], and electromagnetic tomography (EMT) [24]. They have similar mathematical formulations. ERT is used below to introduce the basic principles.

**Figure 1:**
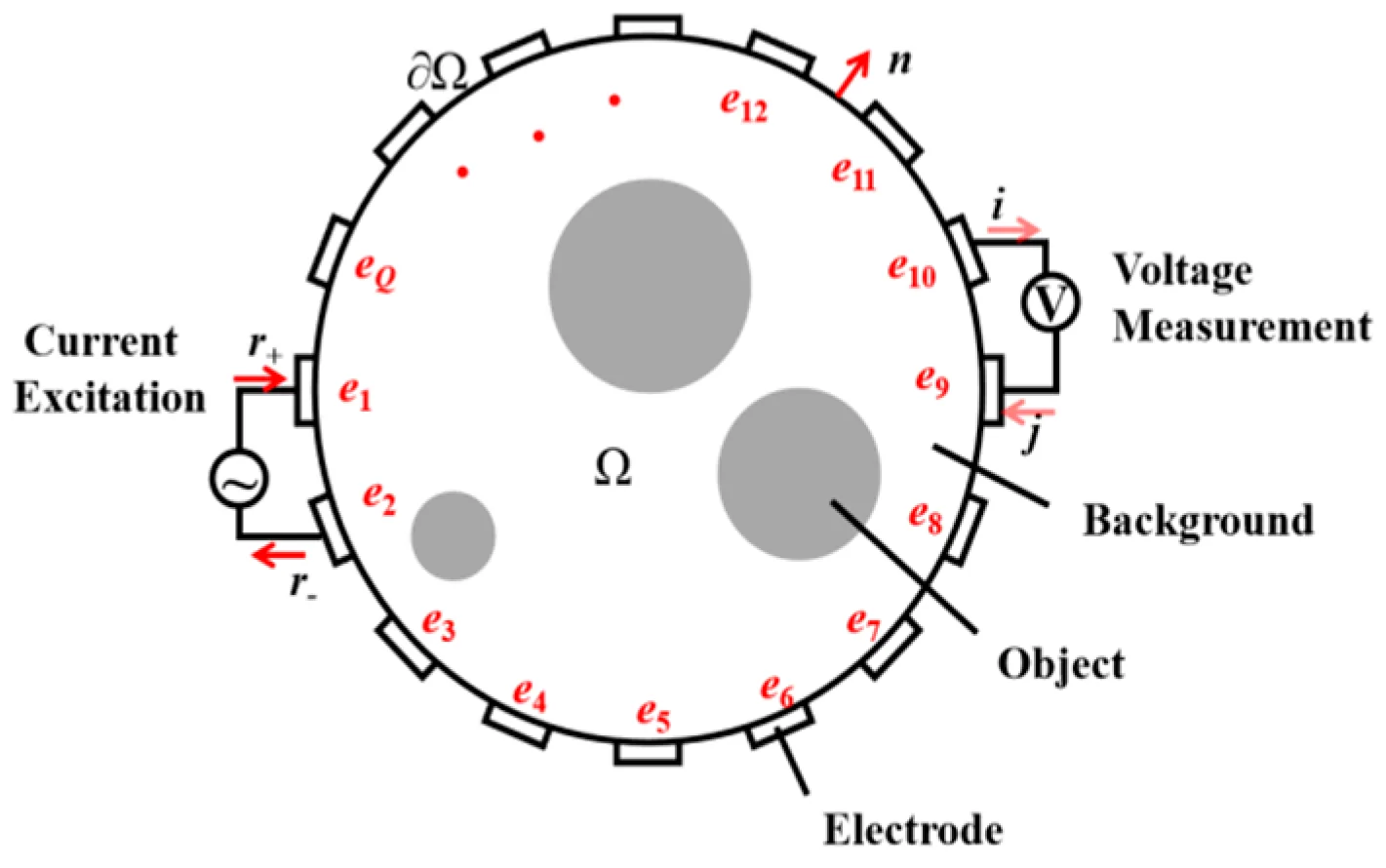
Schematic of the ERT measurement process.

The ERT measurement principle is illustrated in Figure 1, where Ω is a circular detection field bounded by ∂Ω, and *e*_1_, *e*_2_, …, *e_Q_* are *Q* electrodes arranged on *∂*Ω. In a typical acquisition, current is sequentially injected through electrode pairs and boundary voltage measurements are obtained on the remaining pairs on *∂*Ω. The complete set of measurements is then used to reconstruct the conductivity distribution within Ω.

For current excitation, the ERT forward model can be written as [25]

(1) $$\nabla \cdot (\sigma^{*}(r)\nabla \varphi (r;r_{+},r_{-}))=0,\;s.t.,\;\sigma^{*}(r)\frac{\partial \varphi (r;r_{+},r_{-})}{\partial n}=-I(\delta (r-r_{+})-\delta (r-r_{-})),\;r\in \Omega ,\;r_{+},r_{-}\in \partial \Omega$$

where *δ* is the Dirac delta function; *I* is the excitation current applied on the boundary ∂Ω; *n* is the outward unit normal to ∂Ω; *r* is an arbitrary point in Ω; *r*^+^ and *r*^−^ denote an adjacent excitation-electrode pair; *i* and *j* denote a measurement-electrode pair; *φ*(*r*; *r*^+^, *r*^−^) is the potential distribution in Ω produced by excitation at (*r*^+^, *r*^−^); and σ*(*r*) is the conductivity distribution in Ω.

Let Ω_0_ be a reference field with homogeneous conductivity *σ*_0_ over all units. Divide Ω into *N* units (pixels). The conductivity changes from Ω_0_ to Ω are Δσ_1_*(*r*), Δσ_2_*(*r*), …, Δσ_N_*(*r*), and the corresponding *M* boundary measurement changes on *∂*Ω are Δ*u*_1_, Δ*u*_2_, …, Δ*u_M_*. Using the finite-element method, Equation (1) can be discretized and linearized as

Δ*u* = *S*Δ*σ*^∗^(*r*) (2)

where Δσ*(*r*) = [Δ*σ*_1_*(*r*), Δ*σ*_2_*(*r*), …, Δσ_N_*(*r*)]*ᵀ* and *U* = [Δ*u*_1_, Δ*u*_2_, …, Δ*u_M_*]*ᵀ*. *S* is the linear sensitivity matrix that maps the conductivity distribution in Ω to boundary measurements on *∂*Ω [26,27]. It is given by *S* = {*s_ij_*}∈*Rᴹ*ˣ*ᴺ* and satisfies

(3) $$s_{\left(i,j\right)}(r)=-\int_{\Omega }\frac{\nabla u^{0}(r;r_{+},r_{-})}{I_{\left(r_{+},r_{-}\right)}}\cdot \frac{\nabla u^{0}(r;i,j)}{I_{\left(i,j\right)}}\mathrm{dS}$$

where ∇*u*(*i,j*) and ∇*u*(*r*^+^,*r*^−^) are the potential gradients associated with the measurement pair (*i, j*) and excitation pair (*r*^+^, *r*^−^), respectively, and *I*(*i, j*) and *I*(*r*^+^, *r*^−^) are the corresponding excitation-current amplitudes.

ERT reconstruction estimates Δσ*(*r*) from Equation (2) when *S* and Δ*u*(*r*) are known. LBP is one of the most widely used algorithms and is expressed as

Δ*σ*(*r*) = *S^T^*Δ*u, s.t.*, Δ*u*∈*∂*Ω, Δ*σ*(*r*)∈Ω (4)

where *Sᵀ* denotes the transpose of *S*. Thus, the reconstruction accuracy of LBP directly depends on the validity of the sensitivity matrix *S*.

A typical criterion for solving Equation (2) is to minimize the following residual:

min *z* = ||*U-Sσ*(*r*)|| (5)

where ‖·‖ denotes the norm selected for the application.

Because *M*≪*N* and electrical tomography exhibits a diffuse or “soft-field” response [28], solving Equation (5) is severely ill-posed. TR mitigates these problems by adding a penalty term to the objective function:

min *z* = ||*Sσ*(*r*)*-U*||+*λ*||*σ*(*r*)|| (6)

where ||*Sσ*(*r*)*-U*|| is the residual term, ||*σ*(*r*)|| is the penalty term, and λ is the regularization parameter. When ‖·‖ is the Euclidean norm, Equation (6) has the analytical solution

*σ_opt_*(*r*) = (*S^T^S* + *λI*)^−1^*S^T^U* (7)

With an appropriate value of λ, TR provides comparatively high spatial resolution in many EIT applications. The regularization parameter λ must normally be selected using an appropriate criterion; the L-curve is among the most widely used methods [29]. Foundational treatments of finite-element EIT modeling, classical electromagnetic theory, and iterative L-curve-based regularization are provided in [30,31,32].

## 3. Proposed Method

This section first develops the center-residual feedback (CRF) method in the detection field. The difference between fields with and without central targets is investigated and central-residual information is enhanced. Then, the sensitivity of central target positions is enhanced. Both measurement and sensitivity enhancements allow the weak central measurement to participate progressively in the EIT reconstruction process.

### 3.1. Closed-Loop Measurement Extraction by CRF

Let the measurement vector for the detection field Ω containing central targets be *u_cen_* ∈ *R^M^*, that for the field without the central target be *u_nom_* ∈ *R^M^*, the reference vector be *u_ref_* when all units in Ω have homogeneous conductivity *σ*_0_, and the sensitivity matrix be *S* ∈ *R^M^*^×*N*^. Here, *M* is the number of measurements and *N* is the number of units (pixels) in Ω. The measurement differences in the two vectors relative to the reference field are

Δ*u_cen_* = *u_cen_* − *u_ref_* (8)

Δ*u_nom_* = *u_nom_* − *u_ref_* (9)

The following vector is called the CRF and is constructed as

*b*^(0)^=Δ*u_cen_*−Δ*u_nom_* = *u_cen_* − *u_nom_* (10)

**Figure 2:**
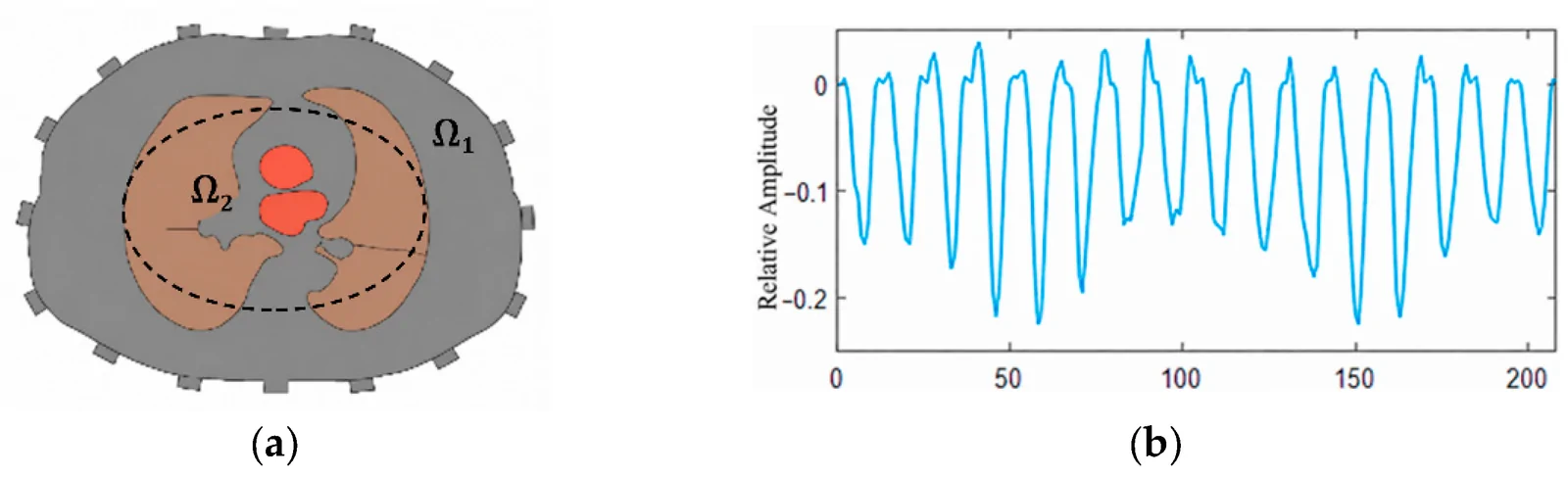
Central-residual measurement absolute and relative differences. (**a**) Detection field with central and boundary targets. (**b**) Relative measurement-difference curve.

It is called the CRF vector caused by central targets in Ω. To evaluate whether the central-residual information is usable, Figure 2a presents a detection field with central and boundary targets. Figure 2b shows the relative difference *b*^(0)^. As seen, these measurement vectors retain a stable and distinguishable difference pattern, and it is distributed across the measurements at low amplitude.

At the reconstruction level, TR is separately applied to Δ*u_cen_* and Δ*u_nom_*, yielding the initial images in Ω with and without the central target:

(11) $$g_{\mathrm{cen}}^{\left(0\right)}=\arg\min_{g}\{||\mathrm{S\sigma}-\Delta u_{\mathrm{cen}}|{|}_{2}^{2}+\lambda ||\sigma |{|}_{2}^{2}\}$$

(12) $$g_{\mathrm{nom}}^{\left(0\right)}=\arg\min_{g}\{||\mathrm{S\sigma}-\Delta u_{\mathrm{cen}}|{|}_{2}^{2}+\lambda ||\sigma |{|}_{2}^{2}\}$$

Applying TR to the initial CRF vector *b*^(0)^ gives the following initial CRF image:

(13) $$\sigma_{\mathrm{cen}}^{\left(0\right)}=\arg\min_{\sigma }\{||\mathrm{S\sigma}-b^{\left(0\right)}|{|}_{2}^{2}+\lambda ||\sigma |{|}_{2}^{2}\}$$

Let the iteration index be *k* = 0, 1, 2, …. At iteration *k*, TR is applied to the vector *b*^(*k*)^ to obtain the CRF image of central targets *g*^(*k*)^:

(14) $$\sigma^{\left(k\right)}={\left(S^{T}S+\mathrm{\lambda I}\right)}^{-1}S^{T}b^{\left(k\right)},\;k=0,\;1,\;2,\;\ldots$$

where λ is set to 0.0010, which is determined by the L-curve method.

Nevertheless, we can obtain the measurement vectors *u_cen_* and *u_ref_*, but *u_nom_* without central targets is usually unavailable. To address the issue, we construct the CRF vector iteratively until a stop condition is met.

Let the detection field Ω be halved into Ω_1_ (boundary subfield) and Ω_2_ (central subfield) (see Figure 2a). The units in Ω_1_ have not only a much larger effect on *u_cen_* than those in Ω_2_ but also larger sensitivity values. Hence, all reconstructed target units in Ω_1_ are denoted as $\sigma_{1}^{\left(0\right)},\sigma_{2}^{\left(0\right)},\ldots ,\sigma_{\left|\Omega_{1}\right|}^{\left(0\right)}$ and taken into Ω in the COMSOL Multiphysics 6.4 simulation platform. After current is injected into the field Ω, the obtained measurements *u*_1_ can initially approximate *u_nom_*. Therefore, the CRF vector is initially computed by

(15) $$b^{\left(1\right)}=u_{\mathrm{cen}}-u_{1}$$

The targets in Ω are reconstructed by the LBP algorithm in terms of *b*^(1)^:

(16) $$\sigma^{\left(1\right)}=S^{T}b^{\left(1\right)}$$

Here, LBP is used because it has no hyperparameters. Repeat the process iteratively using

(17) $$b^{\left(k\right)}=u_{\mathrm{nom}}-u_{k}$$

The process stops when all target units $\sigma_{1}^{\left(k\right)},\;\sigma_{2}^{\left(k\right)},\ldots ,\;\sigma_{\left|\Omega_{1}\right|}^{\left(k\right)}$ in *σ*^(*k*)^ satisfy the following conditions:

(18) $$\sum_{\sigma_{i}^{\left(k\right)},\;\sigma_{j}^{\left(k\right)}\in \Omega_{1},i\neq j}\left|\sigma_{i}^{\left(k\right)}-\;\sigma_{j}^{\left(k\right)}\right|/|\Omega_{1}|<\delta$$

where *δ* is a small positive number. Equation (18) means that the effect of all target units in Ω_1_ on *u_nom_* tends to disappear and $\sigma_{1}^{\left(k\right)},\;\sigma_{2}^{\left(k\right)},\ldots ,\;\sigma_{\left|\Omega_{1}\right|}^{\left(k\right)}$ have nearly homogeneous distributions, and thus *u_nom_* is mainly generated by the central units in Ω_2_.

With the final *b*^(*k−*1)^ as the input to the TR, the final targets in Ω_2_ are reconstructed by

(19) $$\sigma^{\left(k\right)}={\left(S^{T}S+\mathrm{\lambda I}\right)}^{-1}S^{T}b^{\left(k-1\right)}$$

A central candidate image component with a spatial central prior and comparatively high confidence is therefore extracted from *σ*^(*k*)^.

Consequently, the final ERT reconstruction in Ω is

(20) $$\sigma_{\mathrm{CRF}}=\sigma_{\mathrm{nom}}^{\left(0\right)}+\sigma^{\left(k\right)}$$

**Figure 3:**
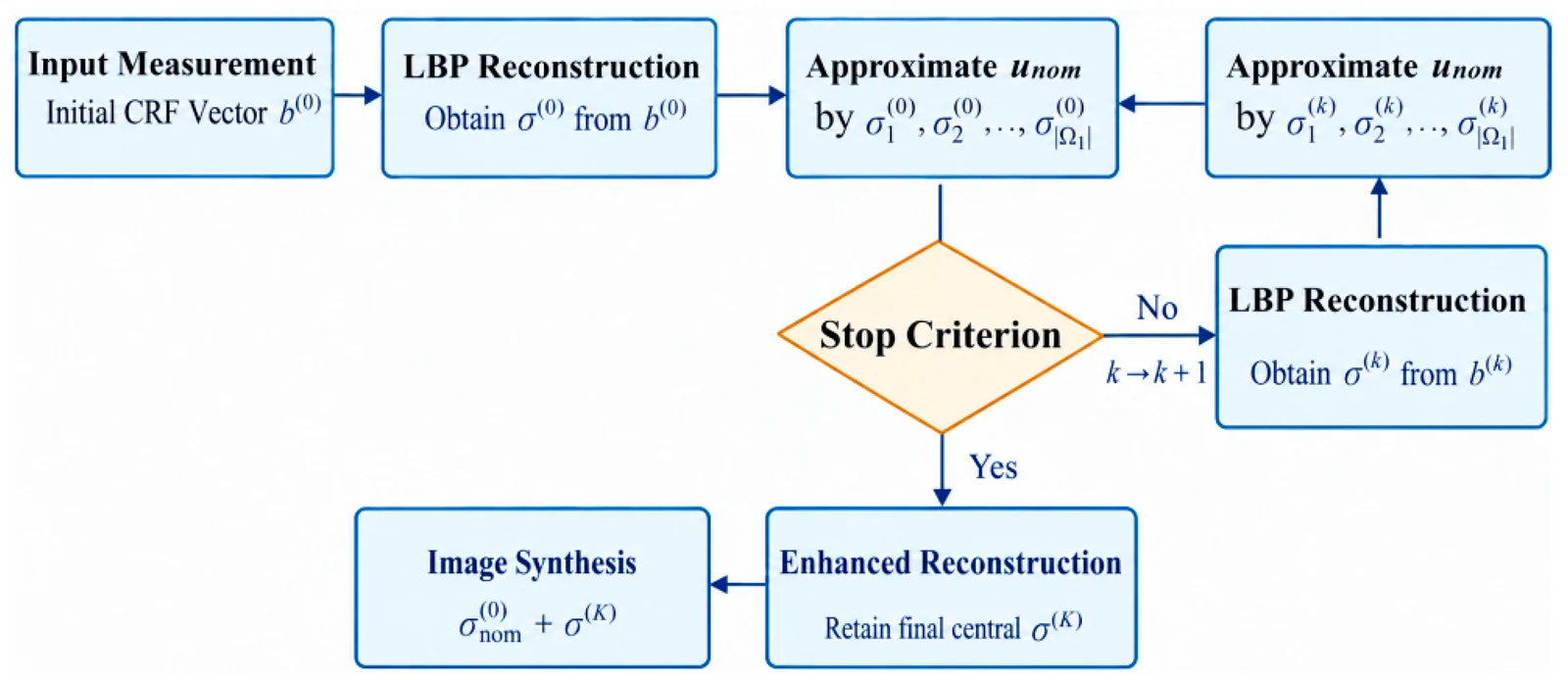
Reconstruction by the CRF vector.

Equation (20) shows that the CRF output is not an isolated central-residual image. Instead, the enhanced central component is superimposed on the baseline structure without the central target, recovering a complete image that contains the central-target information. The resulting closed-loop CRF procedure is illustrated in Figure 3.

### 3.2. Measurement Enhancement by Sensitivity Decomposition

Due to the sensitivity of these units in Ω_1_, *b*^(*k*)^ inevitably contains low-amplitude noise and dispersed artifacts even when Equation (18) is satisfied. They may weaken the ability of *b*^(*k*)^ to reconstruct targets in Ω_2_. To address this issue, like our previous research [21], we further use the following measurement enhancement by decomposing sensitivity coefficients in *S*.

All units in Ω_1_ play a nearly consistent and larger role in *b*^(*k*)^, while those units in Ω_2_ have inconsistent distributions and play a smaller role. Therefore, to enhance the role of all units in Ω_2_, the *k*th CRF vector *b*^(*k*)^ in Ω is decomposed into two new vectors $b_{\Omega_{1}}^{\left(k\right)}$ in Ω_2_ and $b_{\Omega_{2}}^{\left(k\right)}$ in Ω_2_, respectively. With $S_{k}^{\Omega_{1}}$ and $S_{k}^{\Omega_{2}}$ denoting the sums of all sensitivity coefficients in Ω_1_ and in Ω_2_, respectively, the two vectors satisfy

(21) $$\left\{\begin{array}{l} b_{\Omega_{1}}^{\left(k\right)}+b_{\Omega_{2}}^{\left(k\right)}=b^{\left(k\right)}\\ b_{\Omega_{1}}^{\left(k\right)}/b_{\Omega_{2}}^{\left(k\right)}=S_{k}^{\Omega_{1}}/S_{k}^{\Omega_{2}} \end{array}\right.,\;k=1,\;2,\;\ldots ,\;m$$

It shows that *b*^(*k*)^ in Ω in fact is a composite value that consists of the roles of both all units in Ω_1_ and all units in Ω_2._ From Equation (21), $S_{k}^{\Omega_{1}}$ and $S_{k}^{\Omega_{2}}$ can be solved as

(22) $$\left\{\begin{array}{l} b_{\Omega_{1}}^{\left(k\right)}=b^{\left(k\right)}S_{k}^{\Omega_{1}}/(S_{k}^{\Omega_{1}}+S_{k}^{\Omega_{2}})\\ b_{\Omega_{2}}^{\left(k\right)}=b^{\left(k\right)}/(S_{k}^{\Omega_{1}}/S_{k}^{\Omega_{2}}+1) \end{array}\right.,\;k=1,\;2,\ldots ,m$$

**Figure 4:**
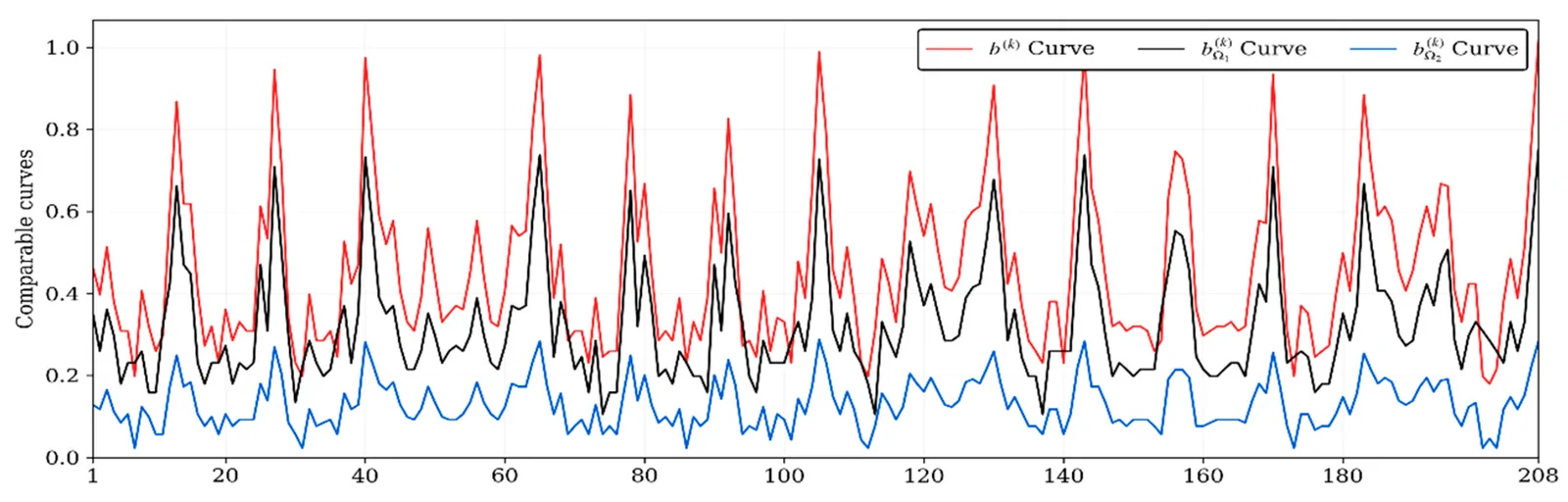
Comparable curves from *b*^(*k*)^, $b_{\Omega_{1}}^{\left(k\right)}$, and $b_{\Omega_{2}}^{\left(k\right)}$.

Figure 4 shows the comparable curves among *b*^(*k*)^, $b_{\Omega_{1}}^{\left(k\right)}$, and $b_{\Omega_{2}}^{\left(k\right)}$. Although the size of $b_{\Omega_{1}}^{\left(k\right)}$ remains larger, the curve of $b_{\Omega_{2}}^{\left(k\right)}$ has a more regular shape, closer to the typical distribution (see Figure 2b), than that of *b*^(*k*)^ since *b*^(*k*)^ is effectively enhanced to $b_{\Omega_{2}}^{\left(k\right)}$. Hence, the value of $b_{\Omega_{2}}^{\left(k\right)}$ can be used to reconstruct the central targets better.

Consequently, the final targets in Ω_2_ are further updated as

(23) $$\sigma^{\left(k\right)}={\left(S^{T}S+\mathrm{\lambda I}\right)}^{-1}S^{T}b_{\Omega_{2}}^{\left(k-1\right)}$$

**Figure 5:**
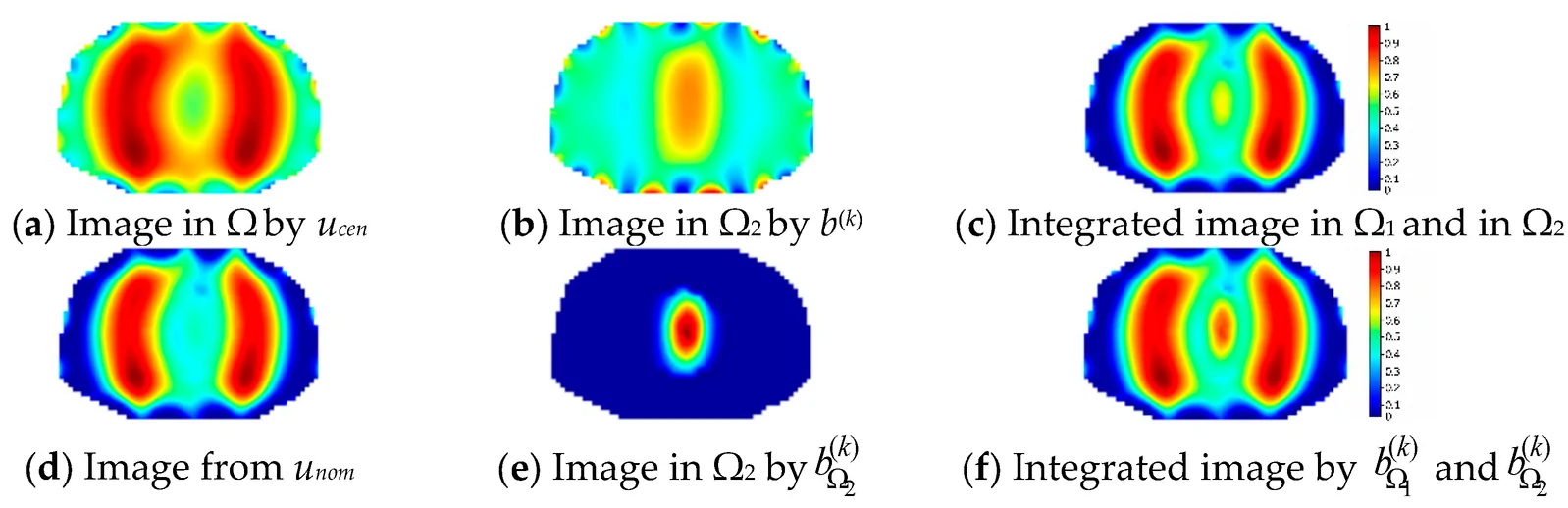
Effect of measurement enhancement using the CRF vector.

According to the model in Figure 2a, Figure 5 presents the CRF vector with and without the measurement enhancement process.

Figure 5a shows the initial image by *u_cen_*, where central targets cannot be found. Figure 5b shows that central targets can be seen in Ω_1_. And Figure 5c shows that their integrated image can reconstruct both boundary and central targets. Alternatively, Figure 5d shows the image by *u_nom_* in which the boundary targets can be reconstructed more clearly, while Figure 5e shows that the central targets can be found. Hence, their integrated image in Figure 5f can reconstruct all targets in Ω more clearly than the image by *u_cen_*.

The joint sensitivity and measurement enhancement method is called JE, and it is summarized in Table 1.

## 4. Results and Discussion

To evaluate the JE method and its iterations, we compare LBP+*b*^(0)^, TR+*b*^(0)^, TR+*b*^(*k*)^, and JE, respectively. The simulations used 16 electrodes, the adjacent-injection pattern, and specified tissue conductivities. We set the model parameters based on the conductivity ratios of heart, lungs, and other soft tissues in a normal adult under 1 MHz excitation, which are 3:1:2.5 at end-inspiration and 5:1:4 at end-expiration, respectively.

The typical correlation coefficient (CC) and relative error (RE) [29] were used as the evaluation metrics.

### 4.1. Simulation Results

**Figure 6:**
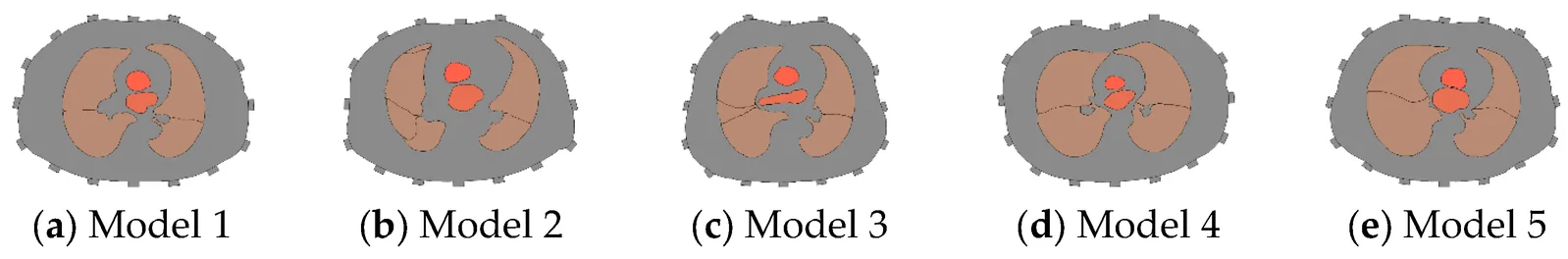
Five cardiopulmonary simulation models.

Thoracic cardiopulmonary simulations and physical experiments were conducted. Five equivalent cardiopulmonary cross-sectional models were selected, as shown in Figure 6. The models differ in lung size, heart position, and thoracic boundary, allowing the JE method to be evaluated under different anatomical configurations.

**Figure 7:**
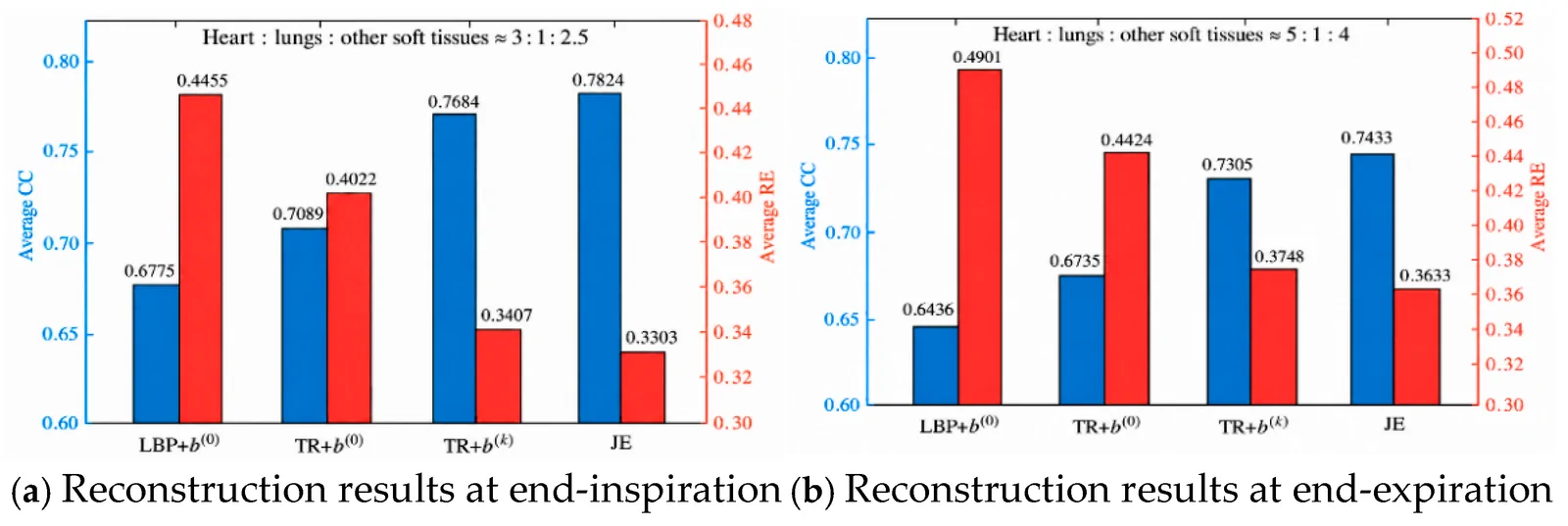
Mean CC and RE for the four methods at (**a**) end-inspiration and (**b**) end-expiration.

Table 2 compares LBP+*b*^(0)^, TR+*b*^(0)^, TR+*b*^(*k*)^, and JE for the five simulated models. The first two methods use the same initial CRF vector and compare the reconstruction algorithms; the third evaluates iterative CRF updating within TR, and JE additionally applies the measurement enhancement. Figure 7 summarizes the mean CC and RE at end-inspiration and end-expiration.

Table 2 shows a consistent improvement from LBP+*b*^(0)^ through TR+*b*^(0)^ and TR+*b*^(*k*)^ to JE: CC increases and RE decreases in every model. Averaging the five model-wise results gives mean CC/RE values of 0.6775/0.4455, 0.7089/0.4022, 0.7684/0.3407, and 0.7824/0.3303, respectively. Relative to LBP+*b*^(0)^, JE increases mean CC by approximately 15.48% and reduces mean RE by 25.87%, calculated before rounding the means. The images show that the initial reconstructions reproduce the broad lung regions but blur or weaken the central heart response. TR+*b*^(*k*)^ and JE make this response more distinct while retaining the bilateral lung structure.

Replacing LBP with TR while retaining *b*^(0)^ produces a modest improvement: mean CC rises from 0.6775 to 0.7089 and mean RE falls from 0.4455 to 0.4022. These changes correspond to a 4.64% CC increase and a 9.71% RE reduction, using the unrounded averages. Regularization improves the reconstruction from the initial measurements, but the central structures remain less clearly separated than in the two methods using iterative feedback. This comparison evaluates the effect of the reconstruction algorithm and should not be interpreted as an isolated sensitivity-enhancement experiment.

Updating the CRF vector from *b*^(0)^ to *b*^(*k*)^ produces the largest successive gain. Compared with TR+*b*^(0)^, TR+*b*^(*k*)^ increases mean CC by 8.38% and reduces mean RE by 15.30%, reaching 0.7684 and 0.3407, respectively. The central response becomes more localized and more clearly separated from the lung regions. Because both methods use TR, this comparison supports the contribution of iterative central-residual feedback to recovering information that is weak in the initial vector.

JE further improves on TR+*b*^(*k*)^, increasing mean CC by 1.82% and reducing mean RE by 3.06%. The gains occur in all five models, although their magnitude depends on the configuration. Model 2 remains the most difficult: JE achieves CC/RE of 0.7258/0.3873, compared with 0.6012/0.5126 for LBP+*b*^(0)^. Model 4 gives the best JE result, 0.8034/0.2675. Visually, JE provides a more concentrated central response than the initial methods, but some smoothing of the true boundaries remains. The results support an additional benefit from the measurement enhancement beyond CRF updating alone.

Figure 7 shows the same method ranking under both conductivity-ratio conditions. At end-inspiration (heart:lungs:other soft tissues ≈ 3:1:2.5), the blue CC bars rise from 0.6775 to 0.7089, 0.7684, and 0.7824, while the red RE bars fall from 0.4455 to 0.4022, 0.3407, and 0.3303. At end-expiration (5:1:4), the corresponding CC values are 0.6436, 0.6735, 0.7305, and 0.7433, and the RE values are 0.4901, 0.4424, 0.3748, and 0.3633. Thus, every method has lower CC and higher RE in panel (b) than in panel (a), indicating poorer reconstruction agreement for the plotted end-expiration condition. Specifically, the end-expiratory and end-inspiratory states represent the two extremes of the entire lung respiratory process. Figure 7 shows that although their reconstruction quality differs, the JE algorithm remains superior. This verifies the effectiveness and generality of JE.

Nevertheless, JE retains the highest CC and lowest RE. The largest successive improvement again occurs between TR+*b*^(0)^ and TR+*b*^(*k*)^, followed by a smaller gain with JE. CC and RE must be read against the left and right axes, respectively; bar heights across the two colors are not directly comparable. Together, Table 2 and Figure 7 support consistent relative benefits across the tested models and the two conductivity ratios.

### 4.2. Experimental Results

**Figure 8:**
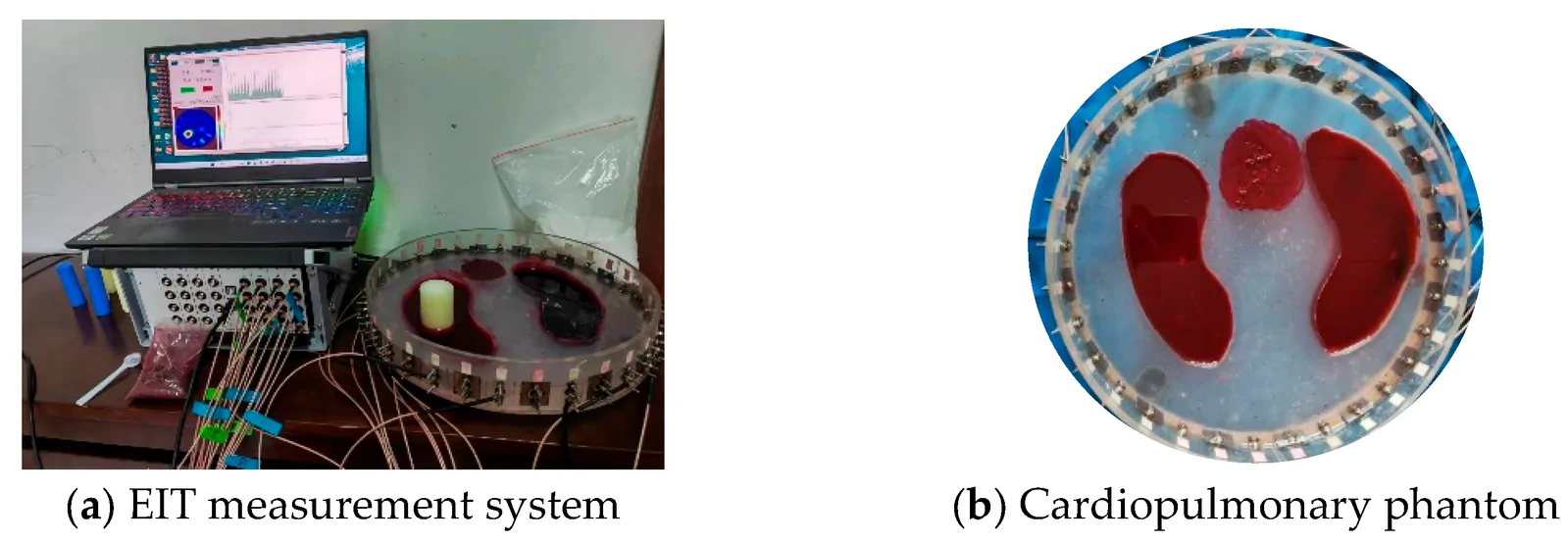
EIT saline-tank cardiopulmonary experimental system.

A saline-tank cardiopulmonary experiment was conducted to evaluate feasibility under practical measurement conditions. Data were acquired using the TJU-EIT II system developed at Tianjin University (Figure 8a). The transparent oblate tank had an inner diameter of 320 mm and a height of 65 mm, with the electrodes positioned 30 mm above the bottom. Agar and saline were mixed and solidified to form the equivalent cardiopulmonary phantom. The background conductivity was 1.55 S/m and the cardiopulmonary conductivity was 2.00 S/m. Rubber rods simulated local abnormalities. Figure 8b and the first column of Table 3 show the experimental models, which contained lung structures, a heart-shaped central object, and abnormalities at different positions.

Table 3 compares LBP+*b*^(0)^, TR+*b*^(0)^, TR+*b*^(*k*)^, and JE for five saline-tank configurations. The columns follow the same order as Table 2, allowing the regularization effects, CRF updating, and additional measuring enhancement to be compared using measured data.

Table 3 reproduces the model-wise ranking observed in simulation: each successive method increases CC and decreases RE in all five configurations. The reported mean CC/RE values are 0.4512/0.6085 for LBP+*b*^(0)^, 0.4682/0.5710 for TR+*b*^(0)^, 0.5350/0.5392 for TR+*b*^(*k*)^, and 0.5490/0.5305 for JE. Compared with Table 2, the experimental CC values are lower and RE values are higher. This loss of reconstruction accuracy is consistent with practical effects such as measurement noise, electrode-contact variation, phantom fabrication differences, and forward-model mismatch; the present comparison does not quantify their individual contributions.

LBP+*b*^(0)^ gives the lowest CC and highest RE in every experimental configuration. Its CC ranges from 0.4348 to 0.4602, while RE ranges from 0.5913 to 0.6164. These values indicate limited agreement with the reference distributions when the initial CRF vector is used directly in back-projection. Configuration 5 has the lowest baseline CC (0.4348), showing that reconstruction difficulty also depends on the arrangement of the experimental targets.

TR+*b*^(0)^ gives a modest improvement over LBP+*b*^(0)^. Using the reported mean values, CC increases by 0.0170 (3.77%) and RE decreases by 0.0375 (6.16%). As both methods use *b*^(0)^, this gain is associated with regularized reconstruction from the same initial vector. The limited change in CC also indicates that regularization alone does not recover all of the information needed to reproduce the experimental structures.

TR+*b*^(*k*)^ provides a larger improvement. Its mean CC of 0.5350 is 18.57% higher than the LBP+*b*^(0)^ baseline, and its mean RE of 0.5392 is 11.39% lower. Relative to TR+*b*^(0)^, mean CC increases by 14.27% and mean RE decreases by 5.57%, based on the reported mean values. The improvements occur in every configuration, supporting the usefulness of iterative CRF updating for measured data. The benefit is therefore not limited to changing the reconstruction solver.

JE achieves the highest CC and lowest RE in all five experimental configurations. From the reported means, its CC increases by 0.0978 (21.68%) and RE decreases by 0.0780 (12.82%) relative to LBP+*b*^(0)^. Compared with TR+*b*^(*k*)^, JE further increases mean CC by 2.62% and reduces mean RE by 1.61%. The model-wise CC gains over TR+*b*^(*k*)^ range from 0.0096 to 0.0176, and the RE reductions range from 0.0054 to 0.0133. For JE, configuration 5 has the lowest CC (0.5248), while configuration 2 has the highest RE (0.5435). The highest CC (0.5620) occurs in configuration 1 and the lowest RE (0.5157) in configuration 3. Thus, the two metrics favor JE consistently while reflecting different aspects of reconstruction agreement.

The simulations and saline-tank measurements both show a modest gain from replacing LBP with TR on *b*^(0)^, a larger gain from iterative CRF updating, and a smaller but consistent additional gain from JE. Experimental errors remain substantial, and the RE reduction relative to the baseline is smaller than in simulation. The results therefore support improved reconstruction accuracy for the tested configurations under practical measurement conditions, without establishing complete target recovery or general robustness to all noise and modeling errors.

## 5. Conclusions

This study proposes a joint enhancement (JE) method for improving the recovery of weak central targets in EIT under adjacent excitation. The method combines center-residual feedback (CRF) with regional sensitivity-based measurement enhancement to increase the contribution of central-target information to reconstruction.

The CRF approximates the unavailable target-free measurements from reconstructed peripheral structures and iteratively updates the residual. Regional sensitivity decomposition then extracts and enhances the measurement component associated with the central subfield. LBP provides rapid intermediate reconstructions, while TR stabilizes the reconstruction of the extracted central component. Integration with the peripheral structure produces a complete image without requiring direct physical measurement of the target-free field.

Across the five simulated models and five saline-tank configurations, JE achieves the highest CC and lowest RE compared with LBP+*b*^(0)^, TR+*b*^(0)^, and TR+*b*^(*k*)^. Relative to LBP+*b*^(0)^, JE increases mean CC from 0.6775 to 0.7824 and reduces mean RE from 0.4455 to 0.3303 in simulation, corresponding to improvements of approximately 15.48% and 25.87%. In the experiments, mean CC increases from 0.4512 to 0.5490 and mean RE decreases from 0.6085 to 0.5305, corresponding to improvements of approximately 21.68% and 12.82%.

These results support improved reconstruction agreement for the tested configurations. Performance nevertheless depends on the accuracy of reference-field estimation and the definition of the central subfield. Future work should assess convergence, false-positive responses, and sensitivity to target position, measurement noise, and model mismatch through controlled tests and independent repeated acquisitions.

## Figures and Tables

**Table 1:** Workflow of the JE method.

Input: Measurement Vector and Sensitivity Matrix *S*.
Output: Integrated EIT image by the CRF-enhanced vector in Ω.
Steps: (1) Compute the measurement difference *u_cen_* for the central targets from *u_ref_*; (2) Construct the CRF *b*^(0)^ from *u_cen_*; (3) Reconstruct all targets by TR in Ω; (4) Approximate *u_nom_* in COMSOL by incorporating all reconstructed targets in Ω_1_; (5) Iteratively apply LBP by *b*^(*k*)^ to obtain *σ*^(*k*)^; (6) If the convergence criterion of Equation (18) is satisfied, proceed to the next step. Otherwise, go to Step 2); (7) Enhance *b*^(*k*)^ by Equation (22) and reconstruct *σ*^(*k*)^ by Equation (23); (8) Combine the final enhanced central-residual image by Equation (20).

**Table 2:** Simulation results for LBP+*b*^(0)^, TR+*b*^(0)^, TR+*b*^(*k*)^, and JE.

Reconstruction Method /Ground Truth	LBP+*b*^(0)^	TR+*b*^(0)^	TR+*b*^(*k*)^	JE	
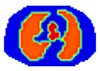	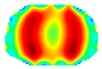	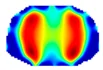	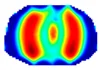	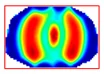	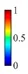
CC/RE	0.6785/0.4532	0.7288/0.3957	0.7849/0.3342	0.8021/0.3296	
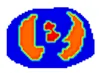	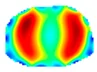	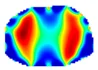	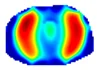	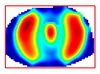	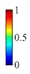
CC/RE	0.6012/0.5126	0.6331/0.4891	0.7008/0.3979	0.7258/0.3873	
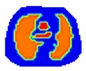	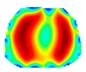	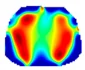	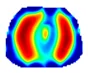	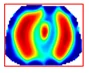	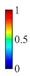
CC/RE	0.6847/0.4456	0.6923/0.4001	0.7791/0.3383	0.7905/0.3301	
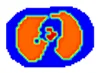	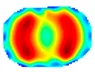	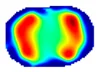	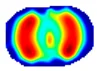	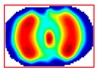	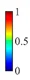
CC/RE	0.7450/0.3543	0.7645/0.3275	0.7981/0.2794	0.8034/0.2675	
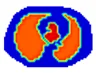	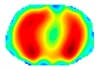	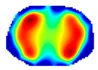	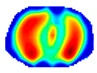	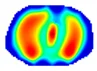	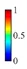
CC/RE	0.6781/0.4619	0.7260/0.3988	0.7790/0.3537	0.7902/0.3369	

**Table 3:** Experimental results for LBP+*b*^(0)^, TR+*b*^(0)^, TR+*b*^(*k*)^, and JE.

Reconstruction Method/ Experimental Model	LBP+*b*^(0)^	TR+*b*^(0)^	TR+*b*^(*k*)^	JE	
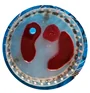	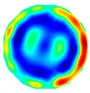	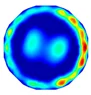	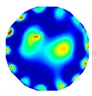	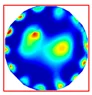	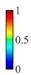
CC/RE	0.4564/0.6143	0.4612/0.5768	0.5499/0.5375	0.5620/0.5266	
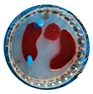	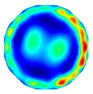	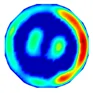	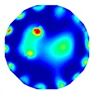	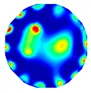	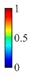
CC/RE	0.4514/0.6164	0.4731/0.5796	0.5363/0.5568	0.5459/0.5435	
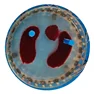	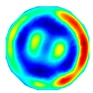	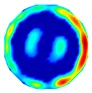	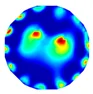	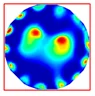	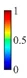
CC/RE	0.4602/0.6077	0.4782/0.5682	0.5423/0.5231	0.5590/0.5157	
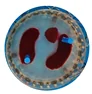	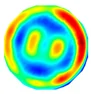	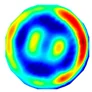	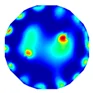	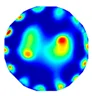	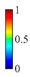
CC/RE	0.4530/0.5913	0.4719/0.5589	0.5391/0.5326	0.5535/0.5262	
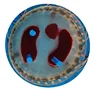	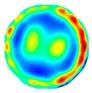	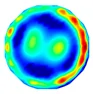	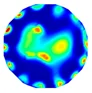	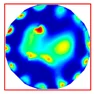	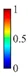
CC/RE	0.4348/0.6126	0.4567/0.5714	0.5072/0.5460	0.5248/0.5406	
Mean CC/RE	0.4512/0.6085	0.4682/0.5710	0.5350/0.5392	0.5490/0.5305	

## Data Availability

The data supporting the findings of this study are available from the corresponding author upon reasonable request, subject to project and industrial data-sharing restrictions.
